# Population-based incidence and demographic patterns of traumatic joint dislocations in children and adolescents

**DOI:** 10.2340/17453674.2025.44880

**Published:** 2025-10-29

**Authors:** William J SÖDERLING, Ilkka J HELENIUS, Petra M GRAHN, Mika V J GISSLER, Topi A LAAKSONEN, Matti M AHONEN

**Affiliations:** 1 University of Helsinki, Faculty of Medicine, Helsinki; 2 Department of Orthopaedics and Traumatology, University of Helsinki and Helsinki University Hospital, Helsinki, Finland; 3 Finnish Pediatric Orthopaedics Research Group (FIPO), Helsinki, Finland; 4 Department of Paediatric Orthopaedics and Traumatology, University of Helsinki and Helsinki University Hospital, Helsinki, Finland; 5 Department of Data and Analytics, Finnish Institute for Health and Welfare, Helsinki, Finland; 6 Academic Primary Health Care Centre, Region Stockholm, Stockholm, Sweden; 7 Department of Molecular Medicine and Surgery, Karolinska Institutet, Stockholm, Sweden

## Abstract

**Background and purpose:**

Limited population-based data is available concerning traumatic joint dislocations in children and adolescents. We aimed to determine the incidence, typical locations, and demographic patterns of joint dislocations in a pediatric population.

**Methods:**

This retrospective, population-based study analyzed pediatric joint dislocations in the Helsinki University Hospital catchment area from 2009 to 2021. Data was retrieved from electronic health records using ICD-10 codes and radiological keywords. Primary radiographs were reviewed to confirm diagnoses. Incidences were calculated using population data, and trends were analyzed by age, sex, and dislocation site. 2,741 traumatic dislocations were included.

**Results:**

The overall annual incidence of joint dislocations was 69 per 100,000 children, with a higher incidence in boys than in girls (72 vs 66 per 100,000, odds ratio 1.1, 95% confidence interval 1.02–1.2). The peak incidence occurred at 16 years of age for boys and 15 years of age for girls. Patellar (49%), elbow (19%), finger (12%), and glenohumeral (10%) dislocations accounted for 90% of cases with respective mean incidence; 35, 13, 8.4 and 7.1 per 100,000.

**Conclusion:**

The annual incidence averaged 69 per 100,000 children. Joint dislocations in children predominantly affect the patellar, elbow, finger, and glenohumeral joints, with adolescence being the most vulnerable period.

Pediatric traumatic joint dislocations are rare and usually occur concomitantly with fractures, presenting rarely as isolated injuries. Joint dislocations are uncommon in young children, as their growth plates are weaker than the ligaments, making fractures more likely than dislocations [1]. Joint dislocations without fractures are more common after growth plate closure [1]. The most frequently dislocated large joints in children are reported to be the patellofemoral, elbow, hip, and glenohumeral joints [2,3].

Single joint dislocations in children have been studied, for example on elbow, patellar, and hip joint dislocations [4-6]. A limited number of studies have examined the population-based incidence of all joint dislocations, including those of the spine and pelvis, in children [4,6-11], but none have systematically characterized joint dislocations in a representative pediatric population.

We aimed to provide a comprehensive, population-based analysis of incidence, typical locations, and demographic patterns of traumatic joint dislocations in children and adolescents.

## Methods

### Design and setting

This is a retrospective population-based register study conducted at Helsinki University Hospital (HUS). HUS New Children’s Hospital is the primary care unit for children residing in the Helsinki metropolitan area providing 24/7 orthopedic care. The hospital utilizes a data lake infrastructure, which stores real-world data generated within the hospital and updates it virtually in real time [12]. The primary catchment area has a population of 1,756,041, including 314,661 children aged 16 years or younger, representing 35% of Finland’s pediatric population (Statistics Finland, December 31, 2023). The age 16 or younger was chosen because in Finland a pediatric patient is someone that is yet to turn 17. We conducted and reported our study in accordance with the STROBE (Strengthening the Reporting of Observational Studies in Epidemiology) guidelines [13].

### Data collection and inclusion criteria

Between 2009 and 2021 ICD-10 codes for traumatic joint dislocations, as well as patient age and sex, were retrieved from the electronic health records of Helsinki University Hospital (HUS), using the data lake infrastructure that contains real-world data generated in the hospital, updated virtually in real time [12]. For this study, relevant ICD-10 codes were combined with a radiological database search using the keywords “fracture,” “dislocation,” and “luxation” to ensure all relevant joint dislocations were included. Only patients aged 16 years and younger residing within the primary catchment area of HUS New Children´s Hospital were included in the study. Data was collected on all joint dislocations, including those of the spine and pelvis, but excluding the temporomandibular joint. Congenital joint dislocations were also excluded, while dislocations accompanied by an avulsion fracture were included. An avulsion fracture is defined as a small piece of bone attached to a tendon or ligament pulled away from the main part of the bone. We included only primary dislocations that occurred during the study period. To ensure complete coverage of all joint dislocations, patients who had a joint dislocation prior to 2009 and sustained a recurrent dislocation during our observation period were included in the study. The following background variables were gathered from the patients: injured joint, age at injury, and sex. There were no missing data on sex or age at injury, as these variables are driven from the personal identity code, which is collected from each patient. Primary radiographs at the time of injury were reviewed to confirm diagnosis of an isolated joint dislocation.

### Statistics

We analyzed time trends of incidences using linear trend estimation, using the test of relative proportions and calculating odds ratios (OR) with 95% confidence intervals (CI) [14]. To evaluate differences over time, we compared the incidence during the last 3 years of the study period (2019–2021) with the first 3 years (2009–2011). The number of children aged between 0 and 16 years during the study period was obtained from the Statistical Yearbooks of Helsinki (City of Helsinki Executive Office, Urban Research and Statistics, 2009–2021, accessed November 15, 2024) to calculate the population-based annual incidence of joint dislocations. Incidence is expressed per 100,000 children (0–16 years).

Sex and age differences were analyzed using the test of relative proportions and Student’s t-test. The age was reported as means with range and standard deviation (SD). To evaluate sex differences, we compared boys with girls by calculating ORs and CIs. Assumptions for statistical tests were evaluated and fulfilled. Level of significance was set to P < 0.05. Statistical analyses were performed by using SAS, version 9.4 (SAS Institute, Cary, NC, USA). Statistical methods used in this investigation have been reported according to SAMPL guidelines. 

### Ethics, funding, and disclosures

The study protocol was approved by the Helsinki University Hospital Review Board (HUS 265/2023). Finnish legislation does not request informed consent from patients or families or ethical committee approval to conduct a purely register-based study. The authors declare no conflicts of interest but received scientific funding from foundations to institution. We received funding from the Finnish Paediatric Research Foundation, Päivikki and Sakari Sohlberg Foundation, Liv och Hälsa Foundation, University of Helsinki Research Foundation for Injuries and Rehabilitation, and Finnish State Funding via HUS Helsinki University Hospital. The funding body was used for researchers’ salaries to conduct this study. Complete disclosure of interest forms according to ICMJE are available on the article page, doi: 10.2340/17453674.2025.44880

## Results

**Figure 1:**
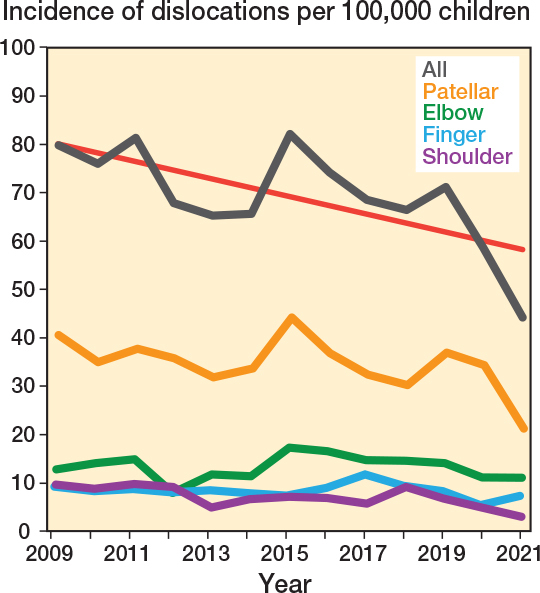
Incidence of the 4 most common joint dislocations per 100,000 children is displayed as pillar charts for the overall rate and the 4 most common dislocations (representing 89% of all cases), with a linear trend (red) line showing the decreasing incidence over time.

2,741 (1,463 boys) patients with a traumatic joint dislocation fitting the inclusion criteria were identified. The mean number of children aged 0 to 16 years residing in the catchment during the study period was 305,299 (SD 7,171, range 295,376–313,479).The mean annual incidence of all joint dislocations was 69 per 100,000 (SD 10, range 44–82) with a significantly lower incidence of most dislocations during the last 3 years of the study (58/100,000) compared with the first 3 (79/100,000, OR 0.9, CI 0.8–0.97, P = 0.01) (Figure 1, Table 1). The difference in incidence between the last and the first 3 years of the study was –20.9 (CI –28.5 to –13.5) (Table 2).

**Table 1:** Descriptive data and incidence of primary joint dislocations per 100,000 children under 17 years of age, in order of occurrence, between 2009 and 2021 in the primary catchment area of HUS New Children’s Hospital. Data are presented as numbers and percentages (%) or means with standard deviation (SD) and range. Odds ratios (OR) for boys are provided with 95% confidence intervals (CI)

Site of dislocation	Patients	Mean age	Boys	Mean incidence	OR boys
	n (%)	(SD) [range]	n (%)	per 10^5^ (SD) [range]	vs girls (CI)
All	2,741 (100)	13 (0.9) [0–16]	1,463 (53)	69 (10.3) [44–82]	1.1 (1.02–1.2)
Patellar joint	1,337 (49)	14 (0.6) [0–16]	557 (42)	35 (5.6) [21–44]	0.7 (0.6–0.8)
Elbow joint	509 (19)	9.4 (1.0) [0–16]	274 (54)	13 (2.5) [7.8–17]	1.1 (0.9–1.3)
One or many finger joints	323 (12)	12 (1.4) [1–16]	221 (68)	8.4 (1.4) [5.4–11.8]	2.1 (1.6–2.6))
Shoulder joint	275 (10)	14 (0.9) [0–16]	200 (73)	7.1 (2.1) [3.0–9.7]	2.6 (2.0–3.3)
Acromioclavicular joint	107 (3.9)	13 (0.9) [0–16]	86 (80)	2.8 (1.1) [0.3–3.4]	3.9 (2.4–6.3)
One or many toe joints	87 (3.2)	12 (1.0) [2–16]	60 (69)	2.3 (0.6) [1.7–3.7]	2.1 (1.4–3.4)
Hip joint	40 (1.5)	12 (1.2) [4–16]	29 (73)	1.0 (0.6) [0–2.0]	2.5 (1.3–5.1)
Tarsometatarsal joint	40 (1.5)	12 (1.3) [0–16]	23 (58)	1.0 (0.8) [0–2.4]	1.3 (0.7–2.4)
Sternoclavicular joint	8 (0.3)	14 (1.2) [11–16]	6	0.2 (0.3) [0.0–0.7]	2.9 (0.6–14)
Knee joint	4 (0.1)	15 (0.3) [13–16]	2	0.1 (0.2) [0–0.3]	1.0 (0.1–6.8)
Talocrural joint	4 (0.1)	13 (0.9) [10–16]	2	0.1 (0.2) [0–0.3]	1.0 (0.1–6.8)
Cervical vertebrae	3 (0.1)	12 (3.4) [5–16]	1	0.08 (0.2) [0–0.7]	0.5 (0.05–5.3)
Wrist and hand joints	3 (0.1)	12 (1.4) [11–16]	2	0.08 (0.1) [0–0.3]	1.9 (0.2–21)
Sacroiliac joint	1 (0.04)	13 (N/A)	–	0.03 (0.1) [0–0.3]	N/A

N/A: not applicable.

**Table 2:** Difference in incidence per 100,000 children between the first (2009–2011) and last (2019–2021) 3 years of the study with 95% confidence intervals (CI)

Site of dislocation	2009–2011		2019–2021		Difference (CI)
	Patients	Incidence	Patients	Incidence	
	n	per 10^5^	n	per 10^5^	
All	701	79	543	58	–20.9 (–28.5 to –13.5)
Patellar joint	336	38	275	29	–8.5 (–13.8 to –3.1)
Elbow joint	123	14	108	12	–2.3 (–5.6 to 1.0)
One or many finger joints	77	8.7	62	6.6	–2.1 (–4.6 to 0.5)
Shoulder joint	83	9.3	44	4.7	–4.7 (–7.1 to –2.2)
Acromioclavicular joint	32	3.6	17	1.8	–1.8 (–3.3 to –0.3)
One or many toe joints	16	1.8	19	2.0	0.2 (–1.0 to 1.5)
Hip joint	13	1.5	8	0.9	–0.6 (–1.6 to 0.4)
Tarsometatarsal joint	15	1.7	5	0.5	–1.2 (–2.1 to –0.2)
Sternoclavicular joint	1	0.1	2	0.2	0.1 (–0.3 to 0.5)
Knee joint	1	0.1	1	0.1	–0.01 (–0.3 to 0.3)
Talocrural joint	1	0.1	1	0.1	–0.01 (–0.3 to 0.3)
Cervical vertebrae	1	0.1	0	0	–0.1 (–0.3 to 0.1)
Wrist and hand joints	1	0.1	1	0.1	–0.01 (–0.3 to 0.3)
Sacroiliac joint	1	0.1	0	0	–0.1 (–0.3 to 0.1)

**Figure 2:**
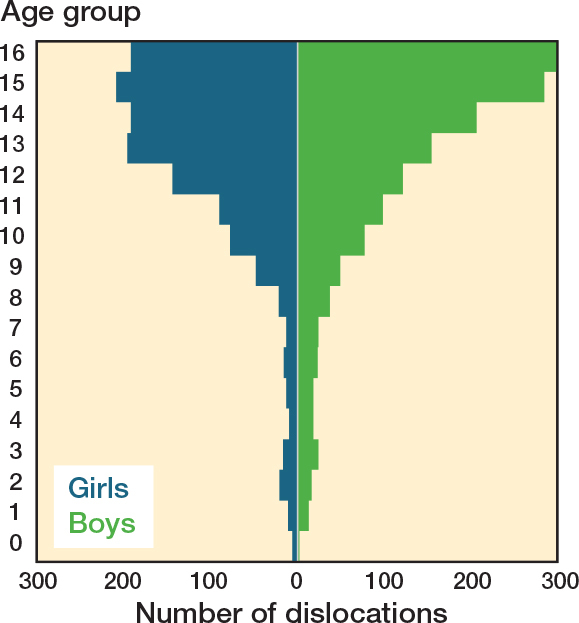
Distribution of joint dislocations by age and sex, showing the number of dislocations (x-axis) for girls (blue) and boys (green) across different age groups (y-axis). The highest number of dislocations occurred at age 15 among girls and at age 16 among boys.

**Figure 3:**
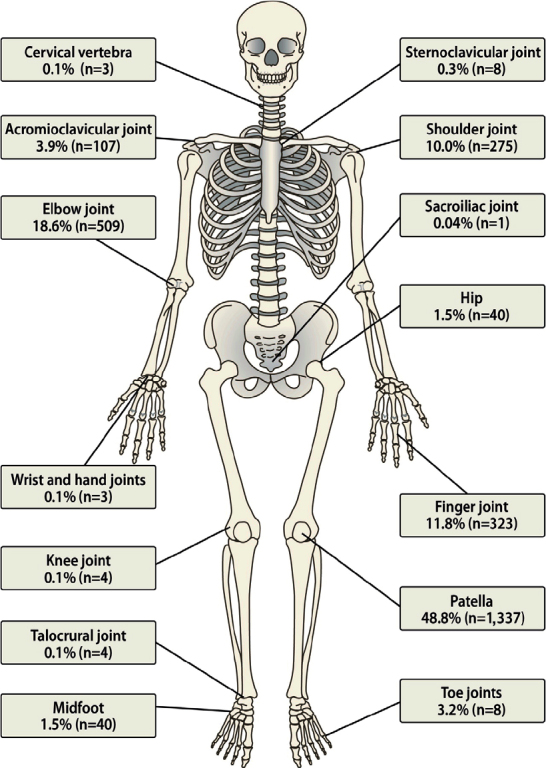
Distribution by joint in number (n) and percentages (%) of the 2,741 primary joint dislocations in children 16 years and younger.

The mean age at traumatic joint dislocation was 13 years (SD 0.9, range 0–16). Joint dislocations were more common in boys (n = 1,463) compared with girls (n = 1,278), respective incidence 72 vs 66 per 100,000 (OR 1.2, CI 1.02–1.2). The incidence of joint dislocations rose exponentially after the age of 8, with a peak in girls at the age of 15, and in boys at 16 (Figure 2). The most common dislocated joint was the patellofemoral joint (n = 1,337, 49%) followed by the elbow (n = 509, 19%). The 4 most common joint dislocations (patella, elbow, glenohumeral, and finger) accounted for 89% of all dislocations in children (see Table 1). The least common sites of dislocations were the lumbar spine, sacroiliac joint, and hemipelvis each occurring in 1 patient each accounting for 1% or less (Figure 3).

The incidence increased only for toe and sternoclavicular joints, but the change did not reach statistical significance (1.8 vs 2.8/100,000, P = 0.2 and 0.1 vs 0.3/100,000, P = 0.3). The difference in incidence between the last and the first 3 years for toe and sternoclavicular joints was 0.2 (CI –1.0 to 1.5) (see Table 2).

### Patellofemoral dislocation

Patellar dislocations represented half (n = 1,337, 48%) of all dislocations recorded during the study period with a mean incidence of 35 per 100, 000 (SD 5.6, range 21–44). The mean age of the patients at time of patellar dislocation was 14 years (SD 0.6, range 0–16). Patellar dislocations were more common among girls (n = 780, mean incidence 40/100,000) compared with boys (n = 557, mean incidence 28/100,000, OR 0.68, CI 0.61–0.76, P < 0.001). The peak incidence of patellar dislocations was observed at age 15 years in both girls and boys (girls 136/100,000, boys 140/100,000). The difference in incidence between the last and first 3 years for patellar joint dislocations was –8.5 (CI –13.8 to –3.1) (see Table 2).

### Elbow dislocation

Elbow dislocation was the second most common (n = 509, 19%) with a mean incidence of 13 per 100,000 (SD 2.5, range 7.8–17). The average age of patients with elbow joint dislocation was 9.4 years (range 0–16) with a sex distribution of 235 girls (12/100,000) and 274 boys (14/100,000, OR 1.1, CI 0.9–1.3, P = 0.2). The peak incidence was at 10 years of age for both sexes (girls 24/100,000, boys 27/100,000). The difference in incidence between the last and first 3 years for elbow joint dislocations was –2.3 (CI –5.6 to –1.0) (see Table 2).

### Finger joint dislocations

Finger joint dislocations (including metacarpophalangeal, proximal, and distal interphalangeal joints) were the third most common (n = 323, 12%). The average age of patients with finger dislocations was 12 years (range 1–16) with a sex distribution of 102 girls (5.3/100,000) and 221 boys (11/100,000, OR 2.1, CI 1.6–2.6, P < 0.001). The peak incidence was at 14 years of age for girls (14/100,000) and at 16 years of age for boys (18/100,000). The difference in incidence between the last and first 3 years for finger joint dislocations was –2.1 (CI –4.6 to –0.5) (see Table 2).

### Glenohumeral joint dislocation

Glenohumeral joint dislocation represented the fourth most common dislocation (n = 275, 10%). The median age of patients with glenohumeral joint dislocation was 14 years (range 0–16) and it was less common in girls (n = 75, 3.9/100,000) compared with boys (n = 200, 9.9/100,000, OR 2.6, CI 2.0–3.3, P < 0.001). The peak incidence occurred at the age of 16 years in both sexes (girls 24/100,000 and boys 69/100,000). The difference in incidence between the last and first 3 years for glenohumeral joint dislocations was –4.7 (CI –7.1 to –2.2) (see Table 2).

## Discussion

This is the first study to describe the population-based incidence, as well as the age and sex distribution, of traumatic joint dislocations in children and adolescents. We aimed to provide a comprehensive, population-based analysis of incidence, typical locations, and demographic patterns of traumatic joint dislocations in children and adolescents. We showed that the incidence of joint dislocations declined significantly over the study period, with the lowest rates recorded in 2020 and 2021. Pandemic-related restrictions likely contributed to this decline by reducing organized sports activities, particularly among pre-teens and teenagers, who are most at risk of joint dislocations. Increased reliance on private healthcare, supported by rising private insurance coverage, may also explain some cases not captured in this study, though this primarily affects minor dislocations not requiring pediatric anesthesia. Even when excluding 2020–2021, the overall trend remains a decline. In a study conducted by the Finnish government in 2023 it was noted that participation in organized sports had been on the decline since 2014, especially in children aged 13–15 years, which is within the peak incidence of most dislocations and could thus further explain the decline in incidence [15].

Patellofemoral dislocations accounted for nearly half of all cases, with a peak incidence at age 15 for both sexes. This aligns with previous studies reporting incidences of 43 and 29 per 100,000, respectively [5,16]. These findings support the observation that acute patellar dislocations are common in pediatric and adolescent populations, with the highest incidence occurring in adolescents and young adults aged 14 to 20 years, likely due to a strong correlation with sports activities [17,18]. Patellofemoral joint dislocations were also more common in girls than in boys. Females have been shown to have greater quadriceps angle, femoral anteversion, and ligamentous laxity than males, all of which are known risk factors for patellofemoral joint dislocation [16].

The second most common dislocation observed was of the elbow joint, where the incidence we found was notably higher than the 6 per 100,000 reported in an earlier Finnish study [4]. This increase aligns with the overall rise in pediatric fractures, particularly forearm fractures, which account for approximately 1/3 of all fractures in children. Elbow dislocations and forearm fractures share a common injury mechanism—most often a fall on an outstretched arm [19,20], which may explain the parallel trends in their incidence. Interestingly, while Hyvönen et al. [4] reported a higher incidence of elbow dislocations in boys (67%), our study found a more balanced distribution between the sexes (46% girls, 54% boys), suggesting potential shifts in activity patterns or risk exposure since the end of Hyvönen’s study in 2019 [4]. Organized sports participation, with approximately 60% of Finnish children involved, may play a role. Girls frequently participate in gymnastics including cheerleading or riding, while boys favor football or ice hockey, all of which are known to predispose to upper limb injuries [4,5,7,15,19,20]. This trend is further supported by previous Scandinavian studies reporting an increase in upper limb fractures [15], likely contributing to the observed rates of elbow dislocations and the third and fourth most common dislocations, those of the finger and shoulder joints.

Rare dislocations in our study included the sternoclavicular, sacroiliac, sacrococcygeal, and cervical vertebrae joints, consistent with previous literature. Sternoclavicular dislocations are uncommon, comprising less than 1% of all pediatric joint dislocations, due to the joint’s strong ligaments, protective anatomy, and the medial clavicular physis’s predisposition to fractures [11]. Sacroiliac dislocations, accounting for 0.3% to 7.5% of pediatric trauma, are typically associated with high-energy impacts like motor vehicle accidents, with fractures being more common than dislocations due to the strong ligamentous support and flexible pelvic anatomy [21]. Cervical vertebrae dislocations are exceptionally rare in children, as ligamentous laxity and horizontally oriented facet joints allow force absorption, though these injuries may result in rare spinal cord injury without radiographic abnormality [22].

### Strengths

A key strength of this study is its population-based design, which provides a comprehensive dataset of pediatric joint dislocations over a 13-year period in children and adolescents aged 16 years and younger. Additionally, the use of primary radiograph reviews to verify diagnoses enhances the accuracy of the findings. For patellofemoral dislocations, joint effusion with or without medial patellar avulsion fracture were interpreted to represent a joint dislocation as these dislocations may typically reduce themselves spontaneously.

### Limitations

As a retrospective, register-based study, the results are subject to potential biases inherent to such designs. While all data was sourced from the electronic health records of Helsinki University Hospital (HUS), some children from the Helsinki area might have been treated elsewhere, potentially affecting the reported incidence rates. Nevertheless, as HUS serves as the referral center for pediatric fractures and joint dislocations, most cases, including follow-up visits after primary reductions performed elsewhere, are likely captured within our dataset. The mechanisms of injury were not available from this large database. Growth zones are not closed in all boys at the age of 16 years, which may limit the interpretation of all adolescent joint dislocations with open physes.

Another limitation is the potential underreporting of minor injuries treated in private clinics, particularly dislocations not requiring on-call pediatric anesthesia. Despite these limitations, the robust design and verification methods employed ensure a high level of reliability in our findings. 

### Conclusion

Traumatic joint dislocations in children and adolescents occurred at an annual incidence of 69 per 100,000, with patellar, elbow, finger, and glenohumeral dislocations accounting for 89% of cases. The incidence peaked in adolescence, with boys and girls showing distinct age distributions. A significant decline in dislocation rates was observed in recent years.

*In perspective,* these findings provide epidemiological insights for both clinical education and the understanding of joint dislocations in pediatric and adolescent populations, which may help in identifying age groups at increased risk and could inform diagnostic considerations for specific types of dislocations.

## Notes

WS: study design, data acquisition, manuscript preparation. IH, MA, PG, TL: study design, manuscript preparation. MG: data analysis, manuscript preparation.

Handling co-editors: Cecilia Rogmark and Philippe Wagner

*Acta* thanks Hanne Hedin and Niels Wisbech Pedersen for help with peer review of this manuscript.
